# Post-transcriptional Processing of mRNA in Neurons: The Vestiges of the RNA World Drive Transcriptome Diversity

**DOI:** 10.3389/fnmol.2018.00304

**Published:** 2018-08-28

**Authors:** Catia Andreassi, Hamish Crerar, Antonella Riccio

**Affiliations:** MRC Laboratory for Molecular Cell Biology, University College London, London, United Kingdom

**Keywords:** neuron, RNA localization, RNA isoforms, 3′UTR, RNA metabolism, translation, RNA processing

## Abstract

Neurons are morphologically complex cells that rely on the compartmentalization of protein expression to develop and maintain their extraordinary cytoarchitecture. This formidable task is achieved, at least in part, by targeting mRNA to subcellular compartments where they are rapidly translated. mRNA transcripts are the conveyor of genetic information from DNA to the translational machinery, however, they are also endowed with additional functions linked to both the coding sequence (open reading frame, or ORF) and the flanking 5′ and 3′ untranslated regions (UTRs), that may harbor coding-independent functions. In this review, we will highlight recent evidences supporting new coding-dependent and -independent functions of mRNA and discuss how nuclear and cytoplasmic post-transcriptional modifications of mRNA contribute to localization and translation in mammalian cells with specific emphasis on neurons. We also describe recently developed techniques that can be employed to study RNA dynamics at subcellular level in eukaryotic cells in developing and regenerating neurons.

## Introduction

**FIGURE 1:**
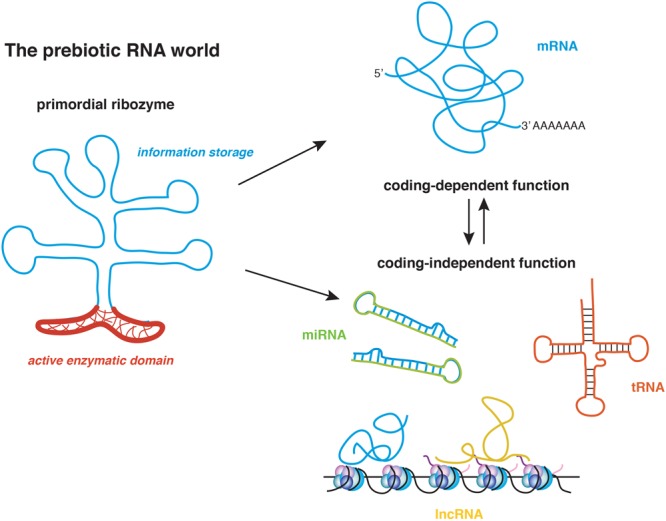
The RNA world postulates that the origin of life coincides with the appearance of the primordial ribozyme, which conjugated coding functions in the nucleotide sequence with enzymatic non-coding functions. This molecule generated the first short polypeptides and through evolution, gave rise to the multifaceted modern RNAs. mRNA, messenger RNA; tRNA, transfer RNA; miRNA, microRNA; lncRNA, long non-coding RNA.

RNA is the most ancient biological polymer whose existence is thought to date back to the prebiotic world. An accredited theory of the origin of life postulates that RNA’s unique ability to couple enzymatic activity with the storage and transfer of information may have triggered the early polypeptide synthesis (**Figure 1**; Pressman et al., 2015). Thus, it is not surprising that as one of the most versatile molecules existing in nature, RNA has been linked to an increasing number of cellular functions in tissues of all organisms. The multifaceted nature of RNA results especially advantageous in complex organs, such as the brain. The mammalian nervous system is composed of several thousands of cells types that must develop and integrate harmoniously to establish functional circuits. The brain must also retain a high degree of flexibility to allow organismal adaptation to the environment. This is achieved through the implementation of gene expression programs that chiefly depend on chromatin accessibility, RNA transcription and translation. Historically, most research on RNA has focused on the open reading frames (ORFs) because of the protein-coding ability. It is now recognized however that the 5′ and 3′ untranslated regions (UTRs) might exert a prominent regulatory role by both contributing to mRNA folding and providing *cis*-elements that recruit RNA binding proteins necessary for transcript localization and translation. mRNA isoforms are generated by alternative splicing, which may include or exclude certain exons, and alternative polyadenylation that generates transcripts with identical ORFs but distinct 3′ UTRs. mRNA can also undergo post-transcriptional base modifications, such as adenosine methylation (Meyer and Jaffrey, 2014; Dominissini et al., 2016; Roundtree et al., 2017) and uridylation (Scheer et al., 2016), which may affect the ability of the transcript to transfer the information encoded by the ORF.

Here, we will discuss some novel aspects of mRNA metabolism and processing with particular emphasis on mechanisms that are relevant to neuronal physiology and will highlight new methodological advances that have allowed the study of cytoplasmic mRNA processing. We refer to a number of recent excellent publications for a comprehensive review regarding the role of the 3′ and 5′ UTRs in mediating RNA localization ad gene expression in various cell types, including neurons (Holt and Schuman, 2013; Mayr, 2017; Costa and Willis, 2018; Sahoo et al., 2018).

## mRNA Isoforms

**FIGURE 2:**
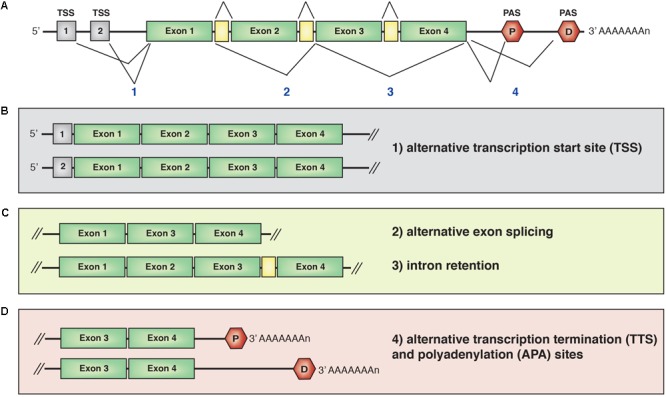
**(A)** Schematic representation of a mRNA transcript. Isoforms can be generated by alternative transcription start site (TTS) choice **(B)**, exon splicing or intron retention **(C)** or alternative transcriptional termination site (TSS) and polyadenylation site (PAS) **(D)**. The combination of two or more mechanisms for the same gene further increases the diversity and number of isoform expression. P, proximal PAS; D, distal PAS.

The expression of protein-encoding genes is controlled by promoters, which are regulatory genomic regions containing sequences around the transcriptional starting sites (TSSs) that recruit transcription factors (Spitz and Furlong, 2012; Slattery et al., 2014; Lambert et al., 2018) and the RNA polymerase II (RNAPolII) machinery (Vo Ngoc et al., 2017). Pre-mRNA transcripts containing a combination of exons and intervening introns are usually very short lived, as they undergo co-transcriptional splicing in a process that removes introns and links exons together (**Figure 2A**). In the nucleus, mRNA is also capped at the 5′ end and polyadenylated at the 3′ end. Alternative spliced transcripts can be generated by the exclusion or inclusion of specific exons. Thus, the same gene can give rise to multiple mRNA isoforms depending on TSS choice, alternative splicing of coding exons or alternative transcriptional termination (TTS) and polyadenylation sites (PAS) choice.

### Alternative Transcriptional Start Sites

Recent genome-wide studies investigated the impact of alternative starting and termination sites on tissue-specific transcriptomes (**Figure 2B**). Analysis of exon usage across 23 cell types using high-throughput 5′ end mRNA sequencing (Reyes and Huber, 2018) combined with Cap Analysis of Gene Expression (Wang et al., 2016) revealed that alternative transcriptional start and termination site choices and alternative splicing of untranslated exons account for most cell type-specific transcriptome. Interestingly, alternative splicing of coding exons generated only 35% of the tissue-specific transcripts. Similar results were previously observed in the cerebellum (Pal et al., 2011). These studies indicate that transcript isoforms are preferentially generated through alternative transcriptional initiation and termination, which principally affect 5′- and 3′-UTRs length, without impacting on the proteome. A potential explanation of these findings is that the strong evolutionary pressure on ORFs has resulted in the synthesis of proteins optimized for certain functions, whereas the UTRs can afford more variability and have diversified widely, giving rise to cell type-specific transcriptomes.

The Brain-derived neurotrophic factor (BDNF) gene is a prototypical example of how multiple TSSs may be exploited to confer distinct functions to a protein, without affecting the polypeptide sequence. BDNF is a small peptide growth factor that regulates neuronal development (Lindsay et al., 1985; Cohen-Cory et al., 2010), synaptic plasticity (Korte et al., 1998) and has been linked to learning and memory (Barco et al., 2005). Dis-regulation of BDNF expression is also associated with a number of neurological disorders, such as epilepsy, autism, Rett syndrome, and Alzheimer’s disease (Zhou et al., 2006; Greenberg et al., 2009; Lu et al., 2013; Hempstead, 2015; Cattaneo et al., 2016). In mouse, the *BDNF* gene has eight 5′ untranslated exons and one exon containing both the translated ORF and the 3′UTR with two alternative polyadenylation sites (Timmusk et al., 1993; Aid et al., 2007). Depending on promoter activation and TSS choice, any of the eight first exons can be used, each one encoding a different 5′UTR. All alternative 5′UTRs are spliced to the common translated exon. The number of BDNF isoforms is further increased by the use of a small alternative intervening exon and in some cases, of different portions of the same 5′UTR exon, which leads to the expression of 11 alternative transcripts. Although the encoded protein is identical in all isoforms, the varied usage of 5′UTRs impacts on the translation rates and cell type-specific expression (Koppel et al., 2015; Cattaneo et al., 2016). Additional *BDNF* isoforms are generated by the alternative usage of polyadenylation sites that give raise to transcripts with either short or long 3′UTRs, affecting differential subcellular localization in response to extrinsic stimuli (An et al., 2008; Will et al., 2013; Vicario et al., 2015).

Another example of the use of alternative TSSs to generate transcripts with distinct 5′UTRs and identical ORF is provided by the gene encoding the neuron-restrictive silencer factor/restrictive element-1 silencing transcription factor (NRSF/REST) (Koenigsberger et al., 2000). NRSF/REST is a nuclear protein that binds and silences neuronal genes in non-neuronal tissues by recruiting epigenetic cofactors to the chromatin (Ballas and Mandel, 2005). Although the gene is widely expressed during early embryogenesis, it is not detected in differentiated neurons where inhibition of NRSF/REST expression is necessary for the transcriptional activation of genes that regulate neuronal functions, including axonal pathfinding and synaptic plasticity. Analysis of the NRSF/REST locus revealed four exons, of which three are promoters that are alternatively spliced to the fourth exon containing the ORF and the 3′UTR (Koenigsberger et al., 2000). Interestingly, promoters were differentially used in neuron-derived cell lines, when compared to fibroblasts and astrocytes. Although little is known regarding the expression of the various isoforms during brain development, it is possible that each promoter responds to specific signal transduction pathways that might regulate *NRSF/REST* transcript levels, localization, and translation.

### Alternative Splicing

Alternative splicing is a nuclear process that takes place co-transcriptionally (**Figure 2C**; Skalska et al., 2017). It has been proposed that at least in some cases, transcripts may be “poised” for splicing in the nucleus before undergoing cytoplasmic remodeling in response to extrinsic stimuli. High-depth RNA sequencing of neocortical tissues at two developmental stages and cortical neurons *in vitro* revealed the presence of retained introns in thousands of polyadenylated transcripts (Mauger et al., 2016). Intron-retaining isoforms are stable and accumulate in the nucleus despite prolonged transcriptional inhibition. Importantly, neuronal activity resulted in excision or increased retention of specific introns. Upon removal of introns, some transcripts were exported to the cytoplasm and loaded onto the ribosomes. These data raise the interesting possibility that post-transcriptional nuclear splicing may represent an additional mechanism to control gene expression, although how neuronal activation influences intron splicing and the impact of the newly translated isoforms on the proteome remain unknown.

Transcripts that retain introns and are exported into the cytoplasm are normally subject to non-sense mediated decay (NMD) and rapidly degraded. NMD is a quality control, RNA surveillance mechanism that ensures the fast removal of transcripts with a premature translation termination codon or long 3′UTRs that may contain introns (Nicholson et al., 2010). Several recent reports have suggested that this may not always be the case and that the presence of retained introns may be part of the physiological processing of mRNA transcripts (Jacob and Smith, 2017). In mouse and rat hippocampal neurons for example, introns are retained in a large proportion of transcripts (44–60%) (Khaladkar et al., 2013). Sequencing experiments indicated that the retained introns harbor *cis*-sequences that contribute to the post-transcriptional regulation of mRNA, including subcellular targeting of the transcripts (Khaladkar et al., 2013). In support of this hypothesis, several intron-retaining transcripts were found in dendrites. Interestingly, many introns contained ID elements, which are Short Interspersed repetitive elements (SINEs) that may retain retrotransposon activity and have a secondary structure similar to the localization signal of *BC1*, a rodent specific non-coding RNA that is transported to dendrites. ID elements induced localization of exogenous sequences to dendrites and competed with endogenous transcripts expressing ID elements, such as the fragile X mental retardation protein *FMRP* for dendritic localization and translation (Buckley et al., 2011).

*Cis*-sequences in retained introns influence RNA localization and translation by interacting with RNA Binding Proteins (RBPs). Staufen2 is a RBP that has been implicated in the regulation of RNA stability (Park et al., 2013). In hippocampal neurons, inhibition of Staufen2 affects the expression levels of only a few interacting mRNAs (Heraud-Farlow et al., 2013), suggesting that it may have additional roles in regulating transcript metabolism. One target of Staufen2 is the retained intron in the 3′UTR of *Calmodulin 3* (*Calm3*) (Sharangdhar et al., 2017). *Calm3* is a calcium-binding protein that acts as an enzymatic cofactor and controls the activity of many proteins, including ion channels. In rat hippocampal neurons, dendritic localization of the *Calm3* isoform carrying the retained intron in the 3′UTR is regulated in an activity-dependent manner. Overexpression of Staufen2 significantly increases the dendritic localization of this specific isoform without affecting mRNA levels (Sharangdhar et al., 2017). In mouse cortical neurons, Staufen2 binds to the retained intron of *CamKIIα* (Ortiz et al., 2017). CamKIIα is a post-synaptic density protein that regulates calcium signaling at glutamatergic synapses and whose activity is required for hippocampal long-term potentiation (LTP) and spatial learning (Hell, 2014). Ortiz and co-workers found that only a small fraction of *CamKIIα* isoforms retaining intron 16 is localized to the cell soma, whereas in dendrites, they represent about one third of total *CamKIIα* mRNA (Ortiz et al., 2017). Binding of Staufen2 to intron 16 stabilizes *CamKIIα* transcript in the cytoplasm and induces dendritic localization. Because neuronal activation causes downregulation of the intron 16-retaining isoform and requires protein translation it is likely that this isoform is subject to NMD. It is unclear however what is the role of the intron retaining isoform and whether degradation is required for the translational activation of the spliced transcript. Intron retention has been observed in the 3′UTR of *Arc* transcript, where regulates dendritic localization (Steward et al., 2017) and the stability of the mRNA (Paolantoni et al., 2018). Interestingly, although the *Arc* transcript expressing the unspliced 3′UTR may be targeted by NMD for degradation, the spliced intron-less isoform is associated with polyribosomes and translated in response to BDNF (Paolantoni et al., 2018).

NMD has also been involved in the local translation of *Robo 3.2* mRNA during the development of commissural axons in mouse (Colak et al., 2013). The Robo 3 gene transcribes two mRNA isoforms *Robo 3.1* and *Robo 3.2*, which is a *bona fide* target of NMD, as it retains an intron with a stop codon located upstream of an exon-junction complex (Black and Zipursky, 2008; Chen et al., 2008). The *Robo 3.1* encoded protein is expressed only prior to axons crossing the midline, after which the protein becomes mostly translated from the *Robo 3.2* isoform. In commissural neurons, *Robo 3.2* transcript is transported to axons following the interaction with the RBP IMP2 (Preitner et al., 2016), but it is kept translationally silent. As the axons cross the midline, the translational inhibition is relieved and local protein synthesis of *Robo 3.2* mRNA is induced. Jaffrey and colleagues found that after translation, *Robo 3.2* mRNA is degraded by the NMD pathway. It should be noted however, that given that NMD is triggered after the first round of translation, it is unlikely that such rapid mRNA degradation may coexist with the robust protein expression necessary for Robo 3.2 accumulation in post-crossing axons. An alternative model postulates that expression of Robo 3.2 proteins is mostly due to the translational inhibition of Robo 3.1. This is based on the observation that *Robo 3.1* and *Robo 3.2* transcripts are expressed at similar levels in the spinal cord at the time of axon midline crossing (Chen et al., 2008).

### Alternative Transcription Termination and Polyadenylation

In eukaryotes, transcription termination of most protein-coding genes takes place concomitantly with endonucleolytic cleavage and polyadenylation of the nascent transcript (**Figure 2D**). The correct execution of this complex process depends on a number of short sequences, such as the hexanucleotide polyadenylation signal (PAS) and other elements located upstream and downstream of the PAS. Most genes carry several PAS signals, and cleavage and polyadenylation can take place at more than one site in a process called alternative polyadenylation. When alternative polyadenylation involves coding exons, it gives rise to multiple mRNA and protein isoforms, whereas alternative choice of PASs located within the 3′UTR generates multiple mRNA isoforms without affecting the protein sequence (Tian and Manley, 2017). Most alternative polyadenylation events occur in exons comprised within the 3′UTR, resulting in the generation of transcripts with 3′UTRs of varied length. This phenomenon has been under positive evolutionary pressure, as the median length and diversity of 3′UTRs increases in higher organisms (Derti et al., 2012). Importantly, 3′UTR complexity is especially relevant in neurons, as they are the cell type expressing the longest and most varied 3′ ends (Hilgers et al., 2011; Ji et al., 2009; Miura et al., 2013; Andreassi et al., 2017; Tushev et al., 2018). These observations support the notion that 3′UTR frequently harbors regulatory elements necessary for most, if not all, aspects of mRNA metabolism, from stability and subcellular localization to translation and degradation (Andreassi and Riccio, 2009; Miura et al., 2014).

A cross-talk between transcription initiation and alternative polyadenylation has been described in *Drosophila* neurons. The nuclear RBP ELAV masks a proximal polyadenylation site causing read-through transcription of RNAPolII and generating mRNA isoforms with longer 3′UTRs (Oktaba et al., 2015). This effect has been linked to the binding of ELAV to gene promoters that contain GAGA elements, which are known to cause RNA PolII pausing. It is not known how ELAV discriminates between proximal and distal PASs, given that ELAV binding sites are not found near the proximal PAS. However, it is possible that additional RBPs may contribute to determine ELAV recruitment to specific PASs. Another example of how 3′ end formation correlates to transcription is provided by hippocampal neurons. In these cells, proximal PAS choice and 3′UTR shortening were linked to activity-dependent expression of MEF2 regulated genes (Flavell et al., 2008). It should be noted however that a recent study performed in hippocampal slices after acute induction of LTP did not find a clear correlation between transcriptional activation and changes in alternative polyadenylation (Fontes et al., 2017). This discrepancy may be due to different model systems and/or paradigms of stimulation used in these studies.

3′UTR formation may also be linked to other post-transcriptional processing events, such as splicing and 5′ capping. A strong link has been observed between expression of the splicing factor U1 snRNP and premature cleavage and polyadenylation (Berg et al., 2012). When U1 snRNP levels are drastically reduced, activation of cryptic intronic PASs near the transcription start site results in premature termination of the nascent transcript within 1 Kb. In contrast, if U1 snRNP expression is only moderately reduced to levels that are insufficient to inhibit the splicing, a switch towards proximal PAS choice and the expression of mRNA isoforms with shorter 3′UTR are observed. It has been proposed that U1 snRNP levels become a critical factor during phases of intense transcription, such as following neuronal activation, possibly leading to 3′UTR shortening found in depolarized neurons (Flavell et al., 2008).

Although the mechanisms leading to differential polyadenylation in neurons are mostly unknown, the relevance to neuronal functions is becoming clear. Alternative polyadenylation plays an essential role in dendritic and axonal localization of many transcripts (Shigeoka et al., 2016; Taliaferro et al., 2016; Andreassi et al., 2017; Tushev et al., 2018), including *BDNF* (An et al., 2008; Will et al., 2013; Vicario et al., 2015), *Impa1* (Andreassi et al., 2010, 2017), *importin β1*(Perry et al., 2012), *mTOR* (Terenzio et al., 2018), and *Shank* (Bockers et al., 2004; Epstein et al., 2014). Localization signals present in isoforms within the 3′UTR usually mediate the transport and activate local translation in response to extrinsic stimuli, such as neurotrophins, synaptic activation and during regeneration after injury. Longer 3′UTRs often harbor miRNA binding sites that decrease protein expression from these isoforms. In the human fetal brain for example, the long 3′UTR of the methyl CpG-binding protein *MeCP2* transcript is targeted and downregulated by miR-483-5p (Han et al., 2013), whereas miR-93, miR-204 and miR-302 bind the long 3′UTR of *Nurr1* and inhibit its expression (Pereira et al., 2017). In human embryonic stem cells (hESCs), *MECP2* transcripts express four tandem 3′UTR variants, but the most abundant isoforms carry either a very short or a very long 3′UTR. Before differentiation into neurons, the *MECP2* isoforms with the long 3′UTR are destabilized by the interaction with Pumilio1 and the binding of miR-200a and miR-302c. In differentiated neurons, miR-200a and -302c expression dramatically decreases, whereas the levels of *MECP2* isoform expressing long 3′UTR and MECP2 protein increase substantially. Translational inhibition is also relieved through a switch of binding of the translation repressor TIA1 with the activator HuC to *MECP2* mRNA (Rodrigues et al., 2016). Thus, while the expression of transcripts with a long 3′UTR could be constitutive, the selective degradation of these isoforms in hESCs may result in an apparent increase in differentiated cells.

### Extranuclear Splicing

In eukaryotic cells, pre-mRNA splicing is executed by the major and minor spliceosome complexes that in addition to sharing common proteins, also contain unique subunits. Minor spliceosome complex-dependent splicing occurs in the cytoplasm, where it affects only a very small fraction (0.5%) of the cellular transcriptome (Turunen et al., 2013). In neurons, subunits of the major spicing complex are detected in the cytoplasm in pathological conditions, such as in patients affected by amyotrophic lateral sclerosis, fronto-temporal dementia and Alzheimer’s disease (Chabot and Shkreta, 2016). Interestingly, in hippocampal neurons, splicing can take place in dendrites severed from the soma (Glanzer et al., 2005) and the splicing factor proline/glutamine rich (SFPQ) contributes to motor neuron axons development in Zebrafish (Thomas-Jinu et al., 2017). In this study, SFPQ promotes motor axon projections cell-autonomously and independently of the nuclear activity by regulating axonal localization of a subset of intron-retaining transcripts. These observations raise the interesting possibility that RNA splicing may take place outside the nucleus. SFPQ has also been linked to neurotrophin-dependent transport of the transcripts *LamininB2, Bcl2/2*, and *Impa1* in rat sensory neuron axons (Cosker et al., 2016). In this case, SFPQ operates independently of the splicing function to assemble structurally related transcripts in RNA granules in a process that promotes axon integrity. Both studies demonstrate that SFPQ coordinates the transport of functionally related transcripts in axons, however whether mRNA is spliced in the cytoplasm to generate isoforms that mediates axon growth and development remains unknown.

## Cytoplasmic Cleavage of 3′Utrs

Cytoplasmic cleavage represents a key step in the biogenesis of small non-coding RNA, including miRNAs and piRNA. The RNAPolII transcribed miRNA precursors are initially remodeled in the nucleus by Microprocessor and subsequently exported to the cytoplasm where they are further cleaved by Dicer, generating two complementary short strands. The strand corresponding to the active miRNA is loaded onto Argonaute (Ago) proteins to form the silencing complex, whereas the other strand is degraded (Bartel, 2018). For mRNA, it is commonly assumed that pre-mRNA is processed in the nucleus and transcripts that escape the nuclear surveillance mechanisms carry signals that target them for cytoplasmic degradation (Nicholson et al., 2010). Thus, cytoplasmic cleavage of mRNA has been generally considered as an integral part of the catabolic process. Capped analysis of gene expression (CAGE) and serial analysis of gene expression (SAGE) (Mercer et al., 2011; Malka et al., 2017) provided initial evidences of 3′UTRs detection independent of the ORFs. Because the 3′UTR fragments do not originate from internal promoters (Mercer et al., 2011) and are not predicted to encode proteins, it has been hypothesized that they could result from a post-transcriptional cleavage process. Recent work from the Hynes group confirmed that for many neuronal transcripts, the coding sequence and 3′UTR of the same mRNA are differentially expressed, implying that these transcripts may undergo cytoplasmic processing (Kocabas et al., 2015). The authors performed Ribo-TRAP sequencing to analyze the transcriptome of developing dopaminergic mouse neurons and noted that for some transcripts there was no uniform reads coverage between 3′UTR and ORF. Further investigations by RT-qPCR and double fluorescent *in situ* hybridization (FISH) confirmed the differential expression of ORF and 3′UTR for many transcripts, including *Sox11* and *Sox12*. The Sox proteins belong to the family of high mobility-group (HMG) domain-containing transcription factors. In mammals, there are more than 20 Sox transcription factors and they influence the expression of downstream target genes necessary for tissue development and cell fate determination. Sox11, 12, and 4 constitute the group C of Sox proteins that despite being closely related, have non-redundant functions during neuronal development (Kamachi and Kondoh, 2013). Transcripts that preferentially express the ORF of a *Sox* transcript in one set of neurons may show prevalent expression of the 3′UTR in a different neuronal type. Importantly, the ORFs and 3′UTRs of several other mRNAs were found to be differentially expressed at various developmental stages and in multiple neuronal tissues. A future challenge will be to understand the role of the 3′UTR fragments and the mechanisms that regulate extra-nuclear processing of mRNA transcripts.

**FIGURE 3:**
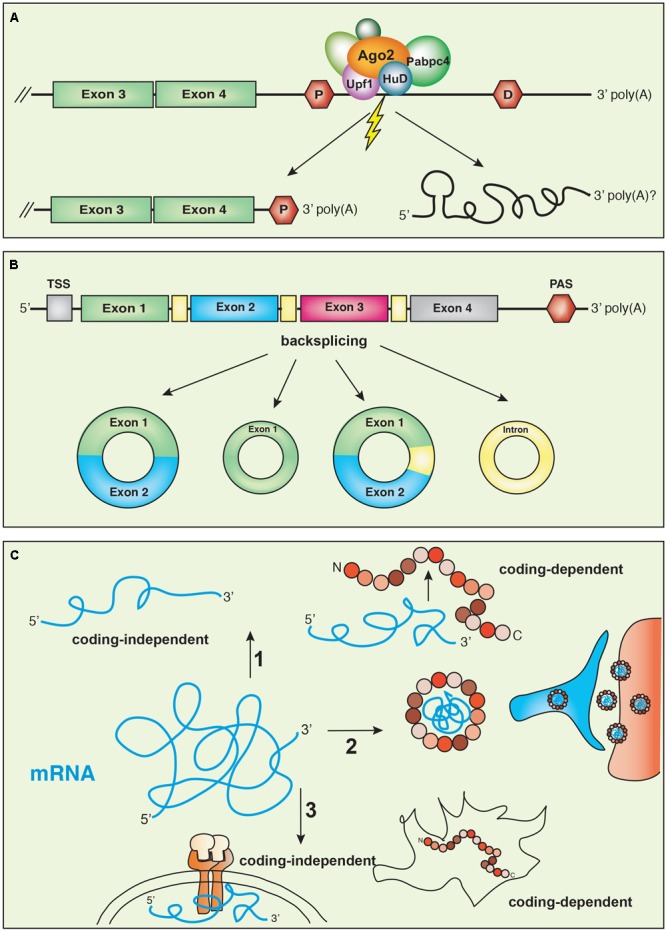
**(A)** 3′UTR remodeling. Transcripts expressing a long 3′UTR can undergo post-transcriptional cleavage and remodeling in response to extrinsic stimuli. The cleavage is mediated by the endonuclease Argonaute2 (Ago2), the helicase UPF1 and the recruitment of the complex to the transcripts involves the RNA binding protein HuD. **(B)** Circular RNAs (circRNAs) are generated by a mechanism of backsplicing that can give rise to transcripts expressing exons, introns or both. **(C)** mRNA transcripts may have multiple functions. (1) In some cases, the same gene encodes coding-dependent and coding-independent isoforms. (2) The *Arc* transcript is translated into a protein that interacts with *Arc* mRNA to form a virus-like structure. (3) The same transcripts may be translated in some cells and have coding-independent functions in others, interacting with membrane receptors, for example.

We recently found that in sympathetic neuron axons, hundreds of 3′UTR may undergo extra-nuclear cleavage, generating isoforms expressing the coding region flanked by a shorter 3′UTR and stable UTR fragments (**Figure 3A**) (Andreassi et al., 2017). Axonal transcripts with short 3′UTRs are generated by cytoplasmic cleavage mediated by a multi-protein complex containing the endonuclease Ago2 and other RNA binding proteins, including Upf1, HuB and HuD, and Pabpc4. We found that the remodeled isoforms originated by the cleavage and expressing the ORF linked to a shorter 3′UTR are more efficiently translated, when compared to the isoforms expressing the same ORF and a long 3′UTR. Therefore, we propose that mRNA transcripts are transported to axons where the 3′UTRs are cleaved possibly in response to extrinsic stimuli. The remodeling of the 3′ end gives rise to isoforms with short 3′UTRs that are translated, and stable 3′UTR fragments of yet unknown function. Importantly, 3′UTR cleavage may not be limited to axons and may be relevant in other physiological contexts. In the rat hippocampus, 3′ end sequencing of RNA isolated from either the soma or the neuropil (that contains only dendrites) revealed extensive differential expression of 3′UTR isoforms following neuronal activation (Tushev et al., 2018). Although in most cases, differential expression of short isoforms was due to transcription rate and/or selective peripheral transport, at least for some transcripts, isoforms with short 3′UTRs were generated by local remodeling of longer 3′UTRs.

What is the fate of the fragments generated by 3′UTR cleavage? It is tempting to speculate that these RNAs produced in axons and dendrites could signal back to inform the cell bodies on the metabolic status of the peripheral compartments. An interesting example of how 3′UTR fragments functionally cooperate with the protein encoded by the parental mRNA has been provided by Vogel and co-workers (Chao and Vogel, 2016). They showed that in *Salmonella enterica*, RNAse E cleaves the 3′UTR of the *CpxP* mRNA, a gene that encodes a protein activated during inner membrane stress. The small RNA released by the cleavage of *CpxP* 3′UTR regulates a number of transcripts that encode proteins involved in the stress response, thereby enhancing CpxP protein functions. Thus, the *CpxP* transcript is cleaved into a protein-coding mRNA and a non-coding 3′UTR fragment that amplifies the regulatory network necessary for the response to stress. In a similar fashion, 3′UTR fragments generated by the cleavage of axonal mRNAs could help to integrate the response of the cell soma to guidance cues and trophic factors applied to axons.

## Circular RNA

Circular RNAs (circRNAs) are RNA molecules derived from precursor mRNAs through a back-splicing mechanism such that the 3′ end of an exon of a protein-coding gene is linked to the 5′ end of an upstream exon (**Figure 3B**; Chen, 2016). Although they were initially considered the result of splicing errors, high-throughput sequencing analyses revealed tissue- and developmental stage-specific expression patterns for thousands of circRNAs (Salzman et al., 2012; Memczak et al., 2013; Guo et al., 2014), suggesting a regulated biogenesis. Because they lack free ends, circRNAs are inherently resistant to exonuclease digestion and are long-lived. Interestingly, their expression can be disjointed from the linear mRNA species from which they derive and in some cases, circRNAs are the major gene product, such in the case of CDR1-as (also known as ciRS-7) (Hansen et al., 2013). The brain expresses the highest number of circRNAs among all tissues and animal species analyzed (Rybak-Wolf et al., 2015), and circRNAs in neurons are enriched at synapses (You et al., 2015). The expression levels are generally very low, although they tend to increase with cell differentiation and during neuronal development (van Rossum et al., 2016). For the circRNAs derived from *Cpsf6, Phf21a*, or *Zfp609* transcripts, the expression does not correlate with the levels of the linear mRNA (Rybak-Wolf et al., 2015). This could be due to the competition of the mechanism for the synthesis of circRNAs with the pre-splicing of the linear pre-mRNA, causing inhibition of the latter. Indeed, this has been demonstrated for circMbl and *muscleblind* mRNA in *Drosophila* (Ashwal-Fluss et al., 2014).

Despite the many evidences of the abundance of circRNAs in the nervous system, their role remains unclear. It has been proposed that circRNAs act as miRNA sponges, competing with other transcripts for miRNA binding. This mechanism has been shown for some circRNAs, including CDR1-as in cell lines (Hansen et al., 2013) and in the mouse and zebrafish brain (Memczak et al., 2013), and for Sry in the testis (Hansen et al., 2013). It should be noted however that CDR1-as represents an exception, as it carries 70 binding sites for miR-7 and can therefore efficiently compete with other miR-7 targets. In most cases, the number of miRNA binding sites per circRNA is quite low (Guo et al., 2014) and it is unclear whether they can efficiently reduce intracellular miRNA levels. A second possibility is that circRNAs function as protein sponges. circMbl for example, harbors several binding sites for the RNA binding protein Muscleblind (Ashwal-Fluss et al., 2014), suggesting that this mechanism may extend to proteins with other metabolic functions that also bind RNA (Castello et al., 2015).

More recently, a new hypothesis of how circRNAs affect cellular functions has been put forward. Translation of circRNAs was previously ruled out due to lack of a significant number of reads in ribosome-profiling studies (Guo et al., 2014; Jeck and Sharpless, 2014; You et al., 2015). This was in contrast to early evidence indicating that many circRNAs contained internal ribosome entry site (IRES) elements and could be translated independently of the 5′ cap (Chen and Sarnow, 1995). Indeed, a subset of circRNAs in *Drosophila* is associated with ribosomes and is translated in a cap-independent manner (Pamudurti et al., 2017). Pamdurti and colleagues generated transgenic flies carrying the circMbl minigene and detected a Mbl immunoreactive band of a size compatible with a polypeptide translated from the transgene (Pamudurti et al., 2017). Interestingly, endogenous circMbl translation was observed in response to starvation, which in flies, activates cap-independent protein synthesis. Similarly, in mammals circZNF609 is associated with polysomes and encodes a peptide that is necessary for myoblast differentiation (Legnini et al., 2017). In neurons, circRNAs are derived by genes encoding large synaptic proteins such as Dscam and Homer1 (Veno et al., 2015) and are enriched at synapses (Rybak-Wolf et al., 2015; Veno et al., 2015; You et al., 2015). An intriguing implication of these findings is that at least some circRNAs may be transported and translated in dendrites in response to synaptic activity.

## Coding-Independent Functions of mRNAs

According to one hypothesis on the origin of life known as the RNA world, RNA was the only multi-functional prebiotic molecule capable of initiating polypeptide synthesis, as it uniquely coupled enzymatic activity with the ability to transfer the information encoded in its sequence (**Figure 1**; Pressman et al., 2015). Thus, it is not surprising that an increasing body of evidence is supporting the idea that RNA transcripts that were once rigidly classified into protein-coding or non-coding RNA (ncRNA) can have multiple and coexistent roles (Nam et al., 2016). ncRNAs include a varied group of transcripts that regulate translation, such as RNA transfer and ribosomal RNAs (tRNAs and rRNAs, respectively), gene expression such as miRNAs and enhancer RNA (eRNA) and chromatin state and transcription, as it is the case for long non-coding RNAs (lncRNAs) (Briggs et al., 2015; Kopp and Mendell, 2018). Yet, the distinction between coding and non-coding transcripts is getting blurred. Many recent studies indicate that protein-coding mRNAs also function in a coding-independent manner (Sampath and Ephrussi, 2016), whereas lncRNAs often harbor short ORFs that encode small peptides (Andrews and Rothnagel, 2014; **Figure 1**).

### mRNA Transcripts With Coding-Independent Functions in Neurons

Examples of coding RNAs with non-coding functions have been described in the nervous system, both in healthy neurons and in pathological conditions (**Figure 3C**; Nam et al., 2016). In rat hippocampal neurons, *Ube3a1*, which is an isoform of the E3 ubiquitin ligase acts as a miRNA sponge, regulating dendritic growth and spine morphogenesis (Valluy et al., 2015). The *Ube3a1* isoform is generated by the activation of an alternative intronic PAS that produces a shorter transcript, which lacks the exons encoding the catalytic centre of the E3 ligase and expresses an alternative 3′UTR. Thus, the protein produced by *Ube3a1* lacks enzymatic activity and is rapidly degraded. Inhibition of *Ube3a1* increases dendritic complexity, spine size and the average amplitude of miniature excitatory postsynaptic currents, whereas silencing of the protein-coding isoform has the opposite effect. Interestingly, *Ube3a*1 effects on dendritic complexity can be rescued by constructs either expressing the full-length *Ube3a1* transcript, the *Ube3a1* 3′UTR sequence or a transcript with a frame shift mutation that cannot be translated. Because 3′UTRs are common targets of miRNA and given that *Ube3a1-*regulated dendritogenesis requires an active miRNA pathway, the authors suggest that the *Ube3a1* transcript compete with other endogenous targets of miR-134 binding. Indeed, silencing of *Ube3a1* in neurons causes a reduction of Limk1 and Pum2, which are both targets of miR-134 (Valluy et al., 2015).

Ubiquitously expressed mRNAs may also function in a non-coding capacity in some cells, while maintaining coding-dependent functions in others. A 3′ end RNA-Seq recently performed in our laboratory (Andreassi et al., 2017), revealed that *Tp53inp2* is one of the most abundant and enriched transcripts in sympathetic neuron axons. Tp53inp2 protein is expressed in myocytes and other cell lines, where it has been linked to autophagy (Sala et al., 2014; Romero et al., 2018). However, despite the abundance of the mRNA, Tp53inp2 protein was not detected in neurons (Crerar and Riccio, unpublished). *Tp53inp2* mRNA structure is unusual in that it comprises a very long 3′UTR (>3000 nt) and a much shorter ORF (600 nt) that predicts a low complex polypeptide without known protein domains. Analysis of a conditional knockout mouse lacking the *Tp53inp2* gene in the sympathetic nervous system revealed loss of sympathetic neurons and axon growth defects at the time when they are highly dependent on the neurotrophin nerve growth factor (NGF) for their development. Surprisingly, we found that although not translated into a protein, the *Tp53inp2* transcript acts in a coding-independent manner by binding to the NGF receptor TrkA and enhancing NGF/TrkA signaling (**Figure 3C**; Crerar and Riccio, unpublished). These findings provide a further layer of complexity to gene regulation and indicate that some mRNA transcripts may function either in a coding or non-coding capacity, depending on the cell type and developmental stage.

### Structural Roles of mRNA Transcripts

Many studies on transcript localization have explored the role of motor and adaptor proteins in mediating the interaction of mRNAs with the intracellular transport machinery (Sahoo et al., 2018). A key unresolved question regards the mechanisms by which RNA transcripts are recognized and sorted by the transport machinery. Few *Cis*-elements that control mRNA localization have been identified (Kislauskis et al., 1994; Andreassi and Riccio, 2009; Merianda et al., 2013), and the general consensus is that the mRNA secondary structure plays a key role in transcript localization. An interesting aspect of the localization process concerns the mRNA export from the nucleus and the nature of the associated proteins that form the ribonucleoparticles (RNP) granules. Molecules that exit the nucleus must cross the Nuclear Pore Complex (NPC), a structure that acts both as a pore and a filter to control nuclear traffic. The size of RNPs largely exceeds the maximum width of NPCs and recently, a novel mechanism based on nuclear egress has been proposed (Parchure et al., 2017). During the infectious cycle, herpes viruses use nuclear egress to export large particles containing the viral dsDNA genome and the capsid proteins from the nucleus of host cells. Viruses usually highjack the cellular mechanisms of the host as cells exploit nuclear egress to transport very large particles, such as RNPs, through the nuclear membrane. Analysis of mRNA contained in large RNPs exported by nuclear egress revealed that 6 out of 19 transcripts encoded synaptic proteins that were locally translated at synaptic sites (Speese et al., 2012), suggesting that nuclear egression may be linked to transcript localization.

Two recent studies demonstrated that for the activity-regulated cytoskeleton associated protein Arc, structure *is* function. Arc is an immediate early gene that is transcribed and targeted to dendrites in response to neuronal activation (Steward et al., 1998). Interestingly, Arc is structurally similar to the group specific antigen (Gag) proteins that are core structural components of retro-viruses, such as the HIV. The Budnik and Thomson group (Ashley et al., 2018) and the Shepherd group (Pastuzyn et al., 2018) discovered that Arc protein can self-assembly into capsid-like structures, forming particles containing *Arc* mRNA that similarly to viruses, are released extracellularly in exosomal vesicles. Importantly, at least in the *Drosophila* neuromuscular junction, *Arc*-particles that are released from axonal presynaptic sites are taken up at the postsynaptic sites in the muscle, where *Arc* mRNA is translated into a functional protein and this process depends on the *Arc* 3′UTR (**Figure 3C**). Thus, transcripts may exploit structural features to switch from coding to non-coding mode. In the case of *Arc*, the mRNA is assembled into virus-like particles to safely navigate the extracellular space only to re-acquire its coding capacity once it has reached the final destination.

## New Technologies to Study mRna Expression in Neurons

Over the last 15 years high-throughput sequencing has revolutionized genomic research. Indeed, the initial evidence for many of the studies discussed here was provided by RNA sequencing experiments. However, cell-population or even single cell sequencing can only provide evidence regarding the expression and relative abundance of mRNA isoforms, but they are rarely used to investigate the subcellular localization and functions of such isoforms. Here, we highlight recent technological developments that allow *in situ* identification, visualization and functional analysis of mRNA isoforms.

**FIGURE 4:**
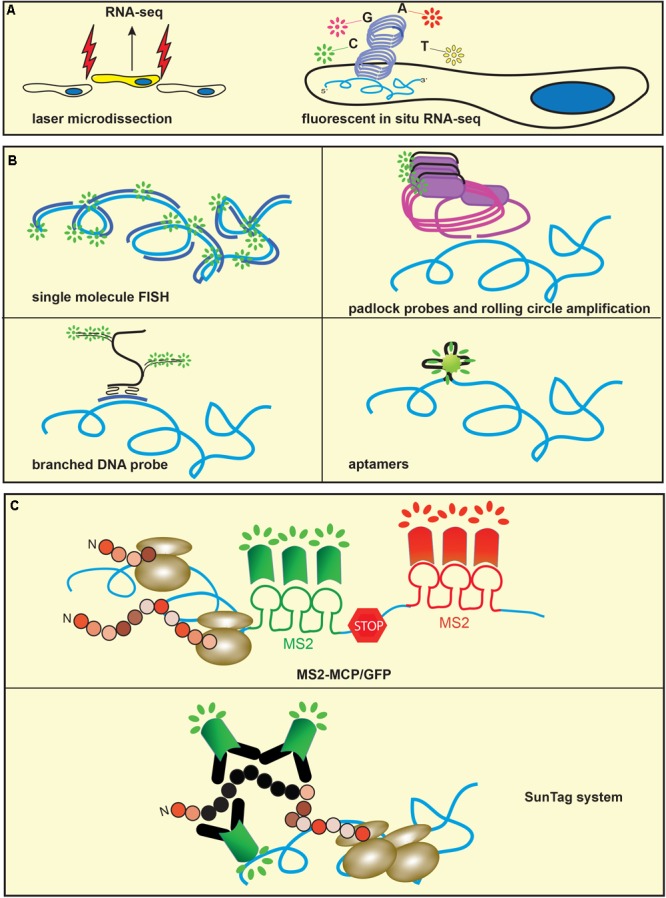
Methods to study mRNA isoform expression and metabolism. **(A)** Sequencing-based methods. Microdissection of specific cell populations (left) or Fluorescent *in situ* RNA Sequencing (right) allows spatially resolved transcriptomics. **(B)** Single molecule FISH (smFISH) and related techniques. Single labeled multiple probes (top left), padlock probes and rolling circle amplification (top right), branched DNA probes (bottom left) or aptamers and related fluorogens, such as Spinach (bottom right) have lowered the threshold of mRNA detection to single molecule. **(C)** Orthogonal systems of RNA stem-loops are engineered in the mRNA of interest and binding proteins are conjugated to fluorescent proteins. The most popular methods to study mRNA transport, local translation and degradation include MS2 stem-loops and MCP-GFP (top) and tandem array peptides engineered in the mRNA of interest and fluorescent single-chain variable fragment antibody (SunTag system, bottom).

To add spatial information to transcriptomic data, RNA-Seq can be combined with ultra-precision dissection of tissues using laser capture microdissection (Farris et al., 2017), microtomy (Kruse et al., 2016) or *in vivo* labeling of specific cell populations (**Figure 4A**; Gokce et al., 2016). These approaches have benefited from the development of a variety of methods of genome-wide amplification of nucleic acids that allow the use of very low amounts of RNA. Sequencing of sections obtained along the Cartesian coordinates of identical samples allows 2D and 3D reconstruction of gene expression maps, providing an unparalleled three-dimensional representation of gene expression (Okamura-Oho et al., 2012; Junker et al., 2014). *In situ* sequencing methods such as rolling circle amplification (RCA) (Mohsen and Kool, 2016) and sequencing by ligation (SBL) (Goodwin et al., 2016) have recently moved transcriptomics to subcellular levels (**Figure 4B**). RCA amplifies circularized probes targeting transcripts of interest to generate concatemers of several copies of the original probe that can be sequenced *in situ* by SBL. This technique employs multiple rounds of ligation, detection and fluorophore cleavage of a pool of differentially labeled oligonucleotides to infer nucleotide sequences. The substitution of target-specific probes with targeted random hexamers is used to obtain *in situ* sequencing of unknown transcripts, allowing the application of this technique to unbiased experimental approaches (Lee et al., 2015).

For many decades, fluorescent *in situ* hybridization (FISH) has been a popular tool for the visualization of transcripts at subcellular levels (Femino et al., 1998). The development of a variety of fluorophores and probes in conjunction with amplification techniques, such as tyramide signal amplification, has allowed the analysis of the subcellular distribution of transcripts in great details. Single molecule FISH (smFISH) is a modification of this technique that takes advantage of a tandem array of short oligonucleotides, all targeting the same transcript and carrying a molecule of fluorophore (Raj et al., 2008). This technical modification has increased the resolution of FISH to single copies of RNA (**Figure 4B**). A more recently developed method for *in situ* visualization of transcripts uses padlock probes and rolling circle amplification. Padlock probes consist of short single strand DNA oligos that carry homology arms complementary to the target sequence and spaced by a linker. Following hybridization of the padlock probe to cDNA, the nick between the homology arms ligates and circularizes the probe that is then amplified to generate thousands of copies of the linker sequence. Fluorescently labeled oligos that bind the linker are used to visualize the amplified product (Krzywkowski et al., 2017). The method relies on the ligation step that occurs only if there is perfect complementarity of the 5′ and 3′ ends of the probe to the target, which allows the detection of single point mutations (**Figure 4B**).

In branched DNA Fluorescence *in situ* hybridization (bDNA FISH), a series of synthetic oligonucleotides probes are hybridized to a short sequence of the target mRNA (Player et al., 2001) and the signal is amplified by a system of preamplifier and amplifier probes (**Figure 4B**). Fluorophore-labeled short oligonucleotides hybridize with the amplifiers, resulting in the visualization of the target mRNA. These techniques have substantially increased the sensitivity and the resolution of FISH so that isoforms originating from alternative splicing (Waks et al., 2011), or single nucleotides changes (Hansen and van Oudenaarden, 2013; Levesque et al., 2013) can be visualized in single cells. A disadvantage of these techniques is the high cost of probes, especially when the analysis is performed on a large number of transcripts. Recently, a method for the enzymatic production of smFISH and RNA capture probes that employs standard molecular biology techniques has been described (Gaspar et al., 2017), making these techniques more accessible to a wider number of research groups.

Although very powerful, mRNA visualization alone is not sufficient to study proteins that interact with transcripts within RBPs and the translational state of the isoforms. To this end, the combination of MS2 RNA motifs with the RNA-binding protein MS2 Coat Protein (MCP) has proved an invaluable tool (**Figure 4c**; Bertrand et al., 1998). The RNA stem-loops derived from the phage MS2 are recognized with high specificity and affinity by MCP, such that if MCP is tagged with a fluorescent protein like GFP, the binding to multiple MS2 sequences located within the 3′UTR of interest allows the detection of single molecules of mRNA. It should be noted however that the presence of several copies of MS2 can affect the metabolism of the targeted mRNA (Garcia and Parker, 2015) or induce aberrant localization of the reporter construct (Heinrich et al., 2017). A recent variation of the technique exploits a reduced affinity of the MS2 binding sites to MCP to allow normal degradation rates of the targeted mRNA (Tutucci et al., 2018). Importantly, this technical approach is suitable for studying mRNA dynamics *in vivo*. Singer and colleagues generated a transgenic mouse by crossing a mouse carrying the MCP-GFP transgene with one in which several copies of MS2 were knocked in into the 3′UTR of the β-actin gene (Park et al., 2014). Remarkably, the authors were able to study *β-actin* mRNA dynamics at very high resolution in brain slices. An alternative tagging system was used by Singer, Park and colleagues to genetically engineer the bacterial protein PP7 binding sites (PBS) into the 3′UTR of the Arc gene (Das et al., 2018). *Arc* transcripts were visualized by a two-color FISH using probes that targeted Arc coding region and PBS. Both transgenic mouse models have essentially confirmed the intracellular dynamics of *β-actin* and *Arc* transcripts that had been observed in studies performed *in vitro*. Strikingly, the authors were able to visualize mRNA subcellular localization in fibroblasts (Park et al., 2014) and dendrites of hippocampal neurons (Das et al., 2018). A potential future development of these techniques will be to investigate the localization and metabolism of specific transcript during neuronal development and in response to complex behaviors in adult mice.

The MS2-MCP system has been used in conjunction with other binary systems like the ones provided by PP7 stem-loops and PP7 protein to visualize transcripts undergoing pioneering round of translation (Halstead et al., 2015) or to study the dynamics of mRNA turnover (Horvathova et al., 2017). These methods however do not allow high temporal resolution of mRNA translation due to the time needed for the synthesis of the fluorescent protein. The introduction of SunTag, a system that uses fluorescently labeled single chain fragments of antibodies expressed by the cells and ready to interact with their target sequences has overcome this issue (**Figure 4C**; Tanenbaum et al., 2014). Finally, short RNA sequences called aptamers that can bind small molecules markedly enhancing their fluorescence have been developed (Bouhedda et al., 2017). Because RNA aptamers and the fluorochrome form a small complex they do not alter the metabolism of the targeted RNA, allowing the detection of the localization and fate of the transcripts. Although these techniques have been developed mostly in cell lines, they can be adapted to the study of isoform expression in neurons, where they will be instrumental to address the relation between RNA structure and proteomic at single cell levels.

## Concluding Remarks

The perception of mRNA is shifting from being a mere intermediate that transfers the genetic information from the DNA into proteins to a signaling molecule with multifaceted functions. Extensive variability of the 5′ and 3′ UTRs has been detected in all tissues and organisms, but it is especially striking in the nervous system. Here, we described the mechanisms that underlie the generation of mRNA isoform diversity and some of the technologies that have allowed an increasingly comprehensive investigation of the transcript repertoire in eukaryotic cells.

Many questions however remain. Although a clear shift in transcript isoform expression has been described in differentiating cells and during brain development, we still do not have a clear picture of the extrinsic signals and the mechanisms that determine specific isoform expression. The issue is further complicated by the findings indicating that identical transcript isoforms may undergo back-splicing to form circRNA or may function in a coding-independent capacity in certain tissues. A future goal will be to perform high throughput screens to gain a comprehensive picture of the percentage of mRNA transcripts that are engaged in translation. A recent study has shown that in retinal axons *in vivo* the translatome changes according to the developmental stage and in adulthood (Shigeoka et al., 2016). Importantly, many mRNA transcripts that are known to be abundant in retinal axons at a certain age are not translated and are “stored”, waiting to be translated at a later time. A potential implication of these findings is that mRNA that is not engaged in translation may have coding-independent functions that contribute to the growth and maintenance of the axons.

Although it was not discussed in this review, reversible post-transcriptional modifications, such as methylation and hydroxymethylation, are adding a further level of complexity to RNA regulation, as they have a profound impact on transcript metabolism and translation (Roundtree et al., 2017). A future challenge will be to understand how nuclear and cytoplasmic processing interacts with chemical modifications of the RNA to generate the transcriptional response that drives organismal development.

The discovery that 3′UTRs are expressed independently of the ORFs (Kocabas et al., 2015) opens a completely new field of research that is not limited to neurons but extends to all organisms, from bacteria to plants and humans. Regulated 3′UTR cleavage of mRNAs (Andreassi et al., 2017) generates an entirely new class of ncRNAs that can be produced either in a constitutive manner or in response to extrinsic stimuli. Although the function of cleaved 3′UTRs is still unknown, the complexity of 3′UTR expression in neurons suggests that this mechanism could be particularly relevant to the nervous system, as numerous cleaved 3′UTR are generated. ncRNA derived from cleaved 3′UTRs are likely to be involved in many neuronal processes, including axon growth and dendritogenesis during the development, and in adult neurons, nerve regeneration, synaptogenesis and synaptic plasticity.

Finally, a view shared by many neurobiologists suggests that neurodegenerative diseases are fundamentally disorders of the RNA (Wang et al., 2007). Thus, defects of the metabolism of the mRNA may be at the core of most, if not all, pathological processes observed in degenerating neurons. The study of the mechanism that regulate mRNA metabolism has the potential to open new therapeutic avenues for the treatment of these highly debilitating and often fatal diseases.

## Author Contributions

CA and AR wrote the manuscript and prepared the figures. HC helped writing the manuscript and designing the figures.

## Conflict of Interest Statement

The authors declare that the research was conducted in the absence of any commercial or financial relationships that could be construed as a potential conflict of interest.
